# Mesenchymal Stromal Cells as Critical Contributors to Tissue Regeneration

**DOI:** 10.3389/fcell.2020.576176

**Published:** 2020-09-25

**Authors:** Georgy D. Sagaradze, Nataliya A. Basalova, Anastasia Yu. Efimenko, Vsevolod A. Tkachuk

**Affiliations:** ^1^Institute for Regenerative Medicine, Medical Research and Education Center, Lomonosov Moscow State University, Moscow, Russia; ^2^Faculty of Medicine, Lomonosov Moscow State University, Moscow, Russia

**Keywords:** mesenchymal stromal cells (MSC), stem cell niche, regenerative medicine, aging, adult stem cells

## Abstract

Adult stem cells that are tightly regulated by the specific microenvironment, or the stem cell niche, function to maintain tissue homeostasis and regeneration after damage. This demands the existence of specific niche components that can preserve the stem cell pool in injured tissues and restore the microenvironment for their subsequent appropriate functioning. This role may belong to mesenchymal stromal cells (MSCs) due to their resistance to damage signals and potency to be specifically activated in response to tissue injury and promote regeneration by different mechanisms. Increased amount of data indicate that activated MSCs are able to produce factors such as extracellular matrix components, growth factors, extracellular vesicles and organelles, which transiently substitute the regulatory signals from missing niche cells and restrict the injury-induced responses of them. MSCs may recruit functional cells into a niche or differentiate into missing cell components to endow a niche with ability to regulate stem cell fates. They may also promote the dedifferentiation of committed cells to re-establish a pool of functional stem cells after injury. Accumulated evidence indicates the therapeutic promise of MSCs for stimulating tissue regeneration, but the benefits of administered MSCs demonstrated in many injury models are less than expected in clinical studies. This emphasizes the importance of considering the mechanisms of endogenous MSC functioning for the development of effective approaches to their pharmacological activation or mimicking their effects. To achieve this goal, we integrate the current ideas on the contribution of MSCs in restoring the stem cell niches after damage and thereby tissue regeneration.

## Introduction

The term “stem cells” refers to the cells that are capable of maintaining their own pool through self-renewal as well as by differentiating into specialized cells of various tissues when certain signals are received. Hence, owing to their functions, stem cells are key participants in morphogenesis, homeostasis, and tissue regeneration (Keyes and Fuchs, 2018). The stem cell pool of an adult organism is predominantly represented by multipotent stem and progenitor cells. They support the structure of tissues and their ability to renew and regenerate throughout life. To become functionally organized, stem cells require a permissive and instructive environment. The pool of adult stem cells, together with its regulatory-specific microenvironment, a stem cell niche, is a functional unit of tissue regeneration. The concept of “stem cell niche” was developed by R. Schofield, who suggested the existence of a microanatomical structure in which the microenvironment determines the behavior of the stem cell. According to this assumption, cells in a niche can proliferate maintaining stemness, and daughter cells that are unable to occupy a niche leave it and differentiate (Schofield, 1978). However, the current conception claims that the coordinated work of multiple components of the niche can not only maintain stem cells but also stimulate the differentiation and return of progenitor cells to an undifferentiated state (Kitadate et al., 2019). Presumably, maintaining stem cells and transferring stem cell properties to niche cells may be an important aspect of damaged tissue regeneration, since they can be recruited to restore tissue. However, the mechanisms for maintaining the stem cell pool may differ from those in homeostasis. Thus, it should be possible to support stem cells even if the structure and function of the niche are significantly altered. So the niche components that harbor these functions must be resistant to tissue damage and activated by damage-associated factors. To ensure balanced regeneration, they must have the ability to perceive a spectrum of local and distant signals of various natures and mediate their transmission to target cells.

In the majority of stem cell niches, mesenchymal stromal cells (MSCs) apparently meet these requirements (Friedenstein et al., 1968; Morrison and Scadden, 2014; Somoza et al., 2016; Degirmenci et al., 2018; Wosczyna et al., 2019). In response to stimuli associated with damage, MSCs can secrete a wide range of extracellular matrix (ECM) components, paracrine factors, and extracellular vesicles (EV), mostly exosomes. When activated, MSCs can also increase their own pool (Itkin et al., 2012) and replenish individual components of the microenvironment by differentiating or attracting supporting cells to a niche. These effects of MSCs are directly or indirectly aimed at maintaining resident stem cells after tissue damage; therefore, MSCs can be considered as a central regulatory component in the regenerating stem cell niche.

## Current Understanding of MSCs

The significant role of stromal cells in regulating the behavior of stem cells was suggested by A. Maximov and later confirmed by A. Friedenstein while investigating hematopoiesis, when it was demonstrated that the hematopoietic microenvironment was created by multipotent bone marrow cells with fibroblast-like morphology, which had the ability to differentiate into the major types of connective tissue cells (Friedenstein et al., 1968; Friedenstein, 1989). These findings were rediscovered and popularized by Owen (1988) and Owen and Friedenstein (1988). Subsequently, this type of cell was termed as the mesenchymal stem cell (Caplan, 1991). At present, multiple studies claim a population of fibroblast-like progenitors is located in the outermost layer of larger arteries and veins, the tunica adventitia (Gomez-Salazar et al., 2020). Importantly, the tunica adventitia possesses many stem cell niche-like characteristics that support and regulate vascular wall progenitor cells including mesenchymal stem cells (Psaltis and Simari, 2015). If further confer properties of mesenchymal stem cells to other tissue-specific stem cells, like skeletal stem cells that generate progenitors of bone, cartilage, and stroma, their niches are localized in fetal and adult bones (Chan et al., 2018). In particular, in mouse model it was found that classical bone marrow MSCs were enriched by leptin receptor (LepR). LepR-positive cells were the main source of CFU-Fs in the bone marrow. It was also shown that these cells gave rise to bone cells as well as adipocytes that formed in adult bone. Moreover, LepR-positive cells were activated for tissue regeneration after irradiation or fracture (Zhou et al., 2014). Since LepR-positive MSCs are localized in close proximity to the sinusoids and arterioles of the bone marrow, it can be assumed that endothelial cells are components of the mesenchymal stem cell niche. This is supported by the presence of extensive crosstalk between MSCs and endothelial cells via PDGFR, BMP, and Notch signaling (Kurenkova et al., 2020).

However, recently, the term “mesenchymal stem cells” was recognized as incorrect due to the accumulated evidence indicating that MSCs did not function in the body only as progenitors for tissues, neither in the normal steady-state nor in disease or injury circumstances (Caplan, 2017). Therefore, the commonly recommended name of these cells now is MSCs, and the presence of multipotent stem cells within MSCs should be carefully evaluated by appropriate assays (Viswanathan et al., 2019).

Till date, cells that meet the minimal MSC characterization criteria (expression of specific surface markers, potential for differentiation into connective tissue cells, and adhesion to plastic) have been found in almost all tissues of the body, and their high prevalence can be attributed to their perivascular localization. However, presumptive MSCs are found notably among pericytes and adventitial cells in the perivascular niche and possibly as interstitial fibroblast-like cells in other compartments. Noteworthy, any fibroblast could fit in MSC definition, however, the relationship between phenotype and cell function may be ambiguous. Thus, we and others have demonstrated in some studies the substantial differences in regenerative effects of so-called MSCs and so-called fibroblasts (Gatti et al., 2011; Basalova et al., 2020).

Despite several common features, MSCs represent a heterogeneous cell population with varied functional and secretory behavior (Melief et al., 2013; Kehl et al., 2019). Direct analysis of perivascular presumptive MSCs has revealed that, within a given tissue or organ, these cells are phenotypically and functionally diverse (Gomez-Salazar et al., 2020). A developmental hierarchy of pericytes and adventitial perivascular cells has been established in human adipose tissue by single-cell transcriptome analysis (Hardy et al., 2017). Correlatively, these two cell types, both of which contribute to conventional cultured MSCs, play distinct roles in osteogenesis *in vivo* (Wang et al., 2019). There is strong evidence indicating the existence of tissue-specific cells, at least in the bone marrow stroma, although with limited ability to differentiate into other cell types (Sipp et al., 2018). The difference between populations of MSCs from different sources is also observed in natural conditions and, apparently, can be persistent, which is confirmed by the weaker osteogenic potential of adipose tissue-derived MSCs, even after osteogenic priming (Brennan et al., 2017). This was also indirectly confirmed by the stable long-term autonomous function of subcutaneous adipose tissue sites during its transplantation to the visceral region (Tran et al., 2008). There are also several other examples of the diverse functional properties of MSCs. Some studies even recommend not using the term MSCs but referring post-natal stem cells to tissue-specific stem cells (such as skeletal or adipose stem cells), which was reflected in the recent International Society of Cell & Gene Therapy (ISCT) recommendations (Viswanathan et al., 2019).

In recent years, a pivotal role of MSCs in the regulation of stem cell niches in various tissues has been intensively explored. The most studied stem cell niche, in which MSCs are key participants in homeostasis and regeneration, is the HSC niche. Thus, MSCs are able to paracrine regulation of the HSC pool by interacting with other cells of the niche and responding to signals from the nervous system (Pinho and Frenette, 2019; Méndez-Ferrer et al., 2020). In the other well-studied niche, a skeletal muscle stem cell niche, MSCs apparently are required for the maintenance of skeletal muscle stem cell pool (Wosczyna et al., 2019). The existence of a perivascular niche for neural stem cells (NSCs) has also been described in the subventricular zone. It has been suggested that MSCs may regulate the local niche by direct contact with NSCs and by secreting different types of neurotrophins, such as BDNF (Somoza et al., 2016). Below we will consider the main mechanisms by which MSCs can participate in the regulation of tissue-specific stem cell niches.

## MSCs are Resistant to Cell Death Signals and Various Damaging Stimuli

AS suggested above, cells that trigger tissue regeneration must be resistant to damage signals and be activated by them. MSCs can be such cells, as they react by activation to the signals of cell death, which are excessively presented in the damaged tissue, or exploit the mechanisms of programmed cell death for survival. Thus, the activation of Fas signaling in MSCs is accompanied not only by apoptosis but also by intensive proliferation, which leads to an increase in the number of them. Presumably, such a response may be the mechanism responsible for their survival under tissue damage and in conditions of inflammation (Solodeev et al., 2018). Similarly, activation of autophagy might protect MSCs from cell death. Particularly, MSCs isolated from transgenic mice depleted for the autophagy proteins BECN1 and LC3B were found to be more sensitive to cell death induced by reactive oxygen species (ROS) than wild-type cells. At the same time, in other cells, autophagy can mediate the turnover of damaged cells (Ghanta et al., 2017).

MSCs can maintain their viability and function through other mechanisms. MSCs express enzymes possessing an antioxidant function and exhibit a high level of glutathione, which can provide resistance to ROS and nitrogen species that accumulate during tissue damage (Valle-Prieto and Conget, 2010). MSCs are also resistant to genotoxic effects. In particular, studies have shown that MSCs display greater viability and lower level of DNA damage than sensitive cells when exposed to cisplatin (Bellagamba et al., 2016) and also demonstrate resistance to radiation damage (Singh et al., 2012). MSCs can also survive and function for a long time under conditions of serum deprivation (Sagaradze et al., 2019b) and hypoxia (Efimenko et al., 2010), which are attributes of *in vivo* ischemic damage.

Therefore, MSCs have a certain resistance to cytotoxic substances that accumulate during damage, as well as to some genotoxic components such as damage inducers. Activation of cell death mechanisms contributes to an increase in the MSC population, which can support tissue regeneration.

## Mechanisms of MSC Participation in Tissue Regeneration

**Figure 1:**
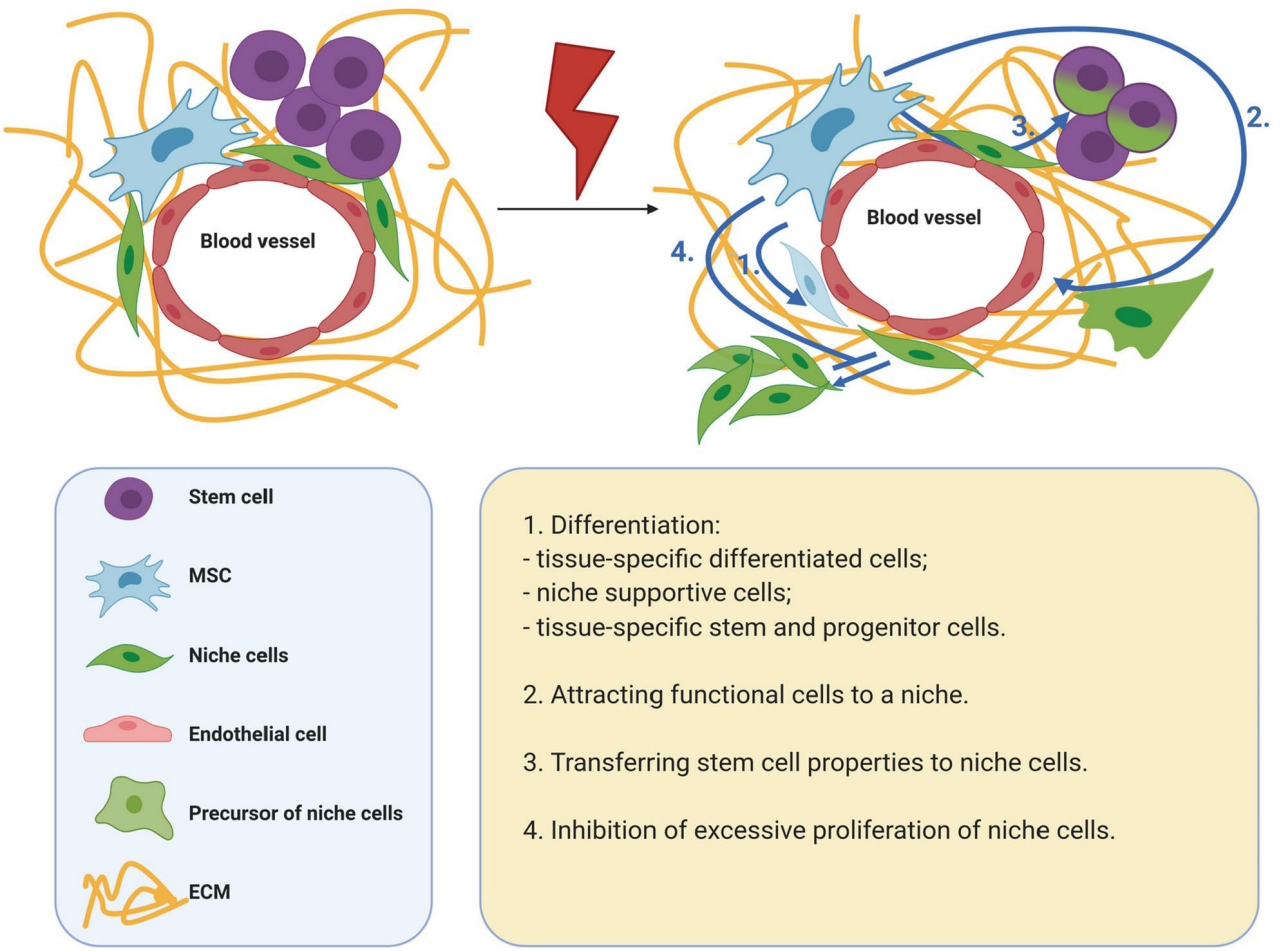
Mechanisms of MSC participation in tissue regeneration. MSCs are able to differentiate into some niche components, as well as attract functional cells to a niche. MSCs are able to replenish the stem cell pool by endowing differentiated cells with stemness. A significant contribution of MSCs to tissue regeneration can be achieved due to their prevalence in tissues and the ability to respond to metabolic, mechanical, biological paracrine stimuli of the microenvironment with high plasticity. MSCs, mesenchymal stromal cells; ECM, extracellular matrix.

For tissue regeneration, it is necessary to preserve the stem cell population and restore the stem cell niche. Presumably, the effects of MSCs can be critical for the implementation of these processes. Thus, to maintain and regulate the behavior of cells in the microenvironment, MSCs secrete many growth factors and cytokines, and are also capable of transferring organelles and extracellular vesicles to target cells. In addition, MSCs actively produce ECM, which has structural and signaling functions. MSCs can also help replenish the cellular composition of the niche. Thus, MSCs are able to differentiate into some niche components, as well as attract functional cells to a niche. In addition, MSCs are able to replenish the stem cell pool by endowing differentiated cells with stemness. A significant contribution of MSCs to tissue regeneration can be achieved due to their prevalence in tissues and the ability to respond to metabolic, mechanical, biological paracrine stimuli of the microenvironment with high plasticity (Figure 1).

### MSCs Regulate Tissue Regeneration at Local and Systemic Levels Through Secretion

It was previously believed that the effects of MSCs are associated with their ability to migrate to the area of damage and differentiate into functional tissue cells. However, the hypothesis that MSCs are involved in tissue regeneration, primarily due to the secretion of growth factors into the intercellular space, was later proposed and confirmed. In a model of acute myocardial infarction, it was demonstrated that secreted MSC products have a cytoprotective effect against cardiomyocytes. The observed effects were achieved in a short time, which additionally reinforced the hypothesis (Gnecchi et al., 2005). Currently, the concept of the important role of the paracrine effects of MSCs in restoring the cellular composition of tissue is generally accepted. Moreover, several researchers have confirmed that paracrine signaling of MSCs is not limited to growth factors and cytokines. In addition, ECM components as well as EV secreted by MSCs can play a significant signaling role.

#### Effects of ECM Components Secreted by MSCs

**Figure 2:**
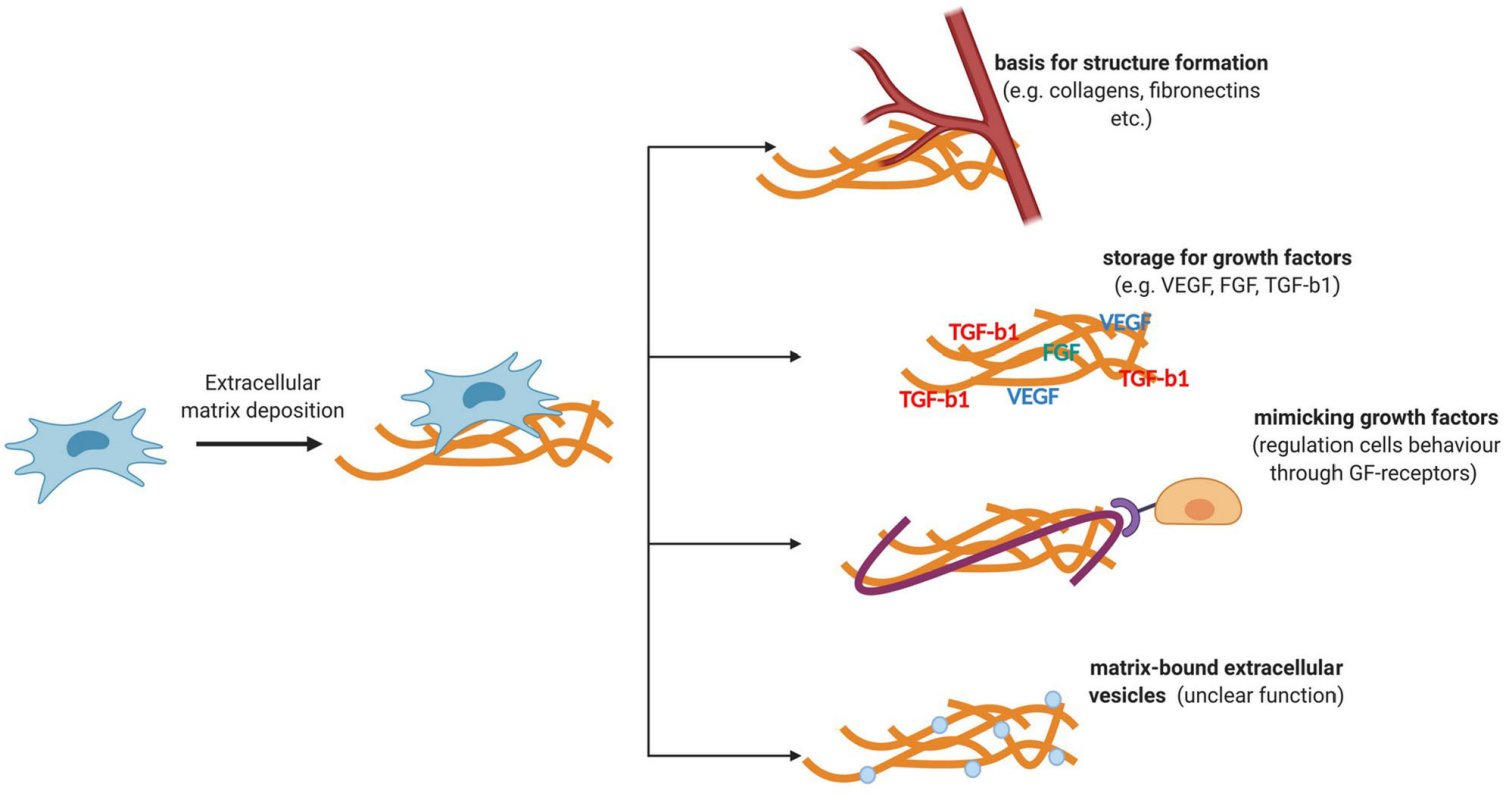
Functions of ECM components secreted by MSCs. ECM is a three-dimensional structure consisting of collagen, fibronectin, elastin, glycosaminoglycans, and various glycosylated proteins that are capable of transmitting mechanical and biochemical cues to cells. ECM stiffness can regulate the behavior of niche cells. The ability of the ECM to transmit biochemical signals is also due to its ability to deposit regulatory molecules and matrix-bounded vesicles, protecting them from degradation and localizing their effects.

The ECM is an important regulatory component of a stem cell niche (Figure 2). It is a three-dimensional structure consisting of collagen, fibronectin, elastin, glycosaminoglycans, and various glycosylated proteins that are capable of transmitting mechanical and biochemical cues to cells. In the stem cell niche, the ECM is involved in the regulation of differentiation, proliferation, and maintenance of the stem cell pool (Novoseletskaya et al., 2019). To fine-tune the signaling function of the ECM, matrix remodeling systems are presented in the niche. Therefore, the supporting cells of the majority of niches secrete matrix metalloproteinases, as well as their inhibitors, which can remodel or maintain a specific ECM structure (Thakkar et al., 2013; Gattazzo et al., 2014; Kalinina et al., 2015a).

Mechanical ECM signals are transmitted to cells through cytoskeletal components and adhesive contacts. Integrins are the key mechanosensors in the cell. The focal adhesion complexes assembled by the participation of integrins turn the mechanical signals of the matrix into biochemical signals (Smith et al., 2017). The nuclear lamina may also be involved in the mechanisms of regulation of certain transcription factors. In particular, the rigidity of the matrix can regulate the expression of lamin A, which can regulate the entry of a transcription factor such as the retinoic acid receptor (Swift et al., 2013) into the nucleus, which, in turn, can affect the differentiation potential of stem cells (Green et al., 2017). Thus, matrix stiffness can regulate the behavior of niche cells. The ability of the ECM to transmit biochemical signals is also due to its ability to deposit regulatory molecules and matrix-bounded vesicles, protecting them from degradation and localizing their effects (Novoseletskaya E. et al., 2019). The abovementioned mechanisms of signal transmission through the ECM can mediate the maintenance of homeostasis and tissue repair.

The ECM components and factors involved in its remodeling are the most represented in the MSC secretome (Kalinina et al., 2015a). MSCs can enhance the secretion of individual ECM components in response to various damage-associated signals. Therefore, in response to vascular damage, MSCs can disconnect from the vascular wall and proliferate and secrete type 1 collagen (Lin et al., 2008). Similarly, the secretion of type 1 collagen and fibronectin by MSCs increases in response to signals regulating wound healing, for example, transforming growth factor beta 1 (TGF-b1) (Desai et al., 2014; El Agha et al., 2017). These structural proteins play a vital role in tissue repair after damage. Hence, type 1 collagen is one of the primary structural and signal components involved in tissue repair and regeneration (Schulz et al., 2015). Fibronectin, in turn, can participate in the transmission of signals to stem cells due to its ability to bind to several molecules, including some of platelet-derived growth factor, vascular endothelial growth factor, fibroblast growth factor families, TGF-b1, and neurotrophin, thus preserving their biological activity.

Furthermore, individual ECM components produced by MSCs can mimic the effects of growth factors by acting as agonists or antagonists of receptor tyrosine kinases. In particular, laminin 5, secreted by MSCs, can bind to the epidermal growth factor receptor and trigger cell differentiation (Klees et al., 2005). In contrast, decorin was found to inhibit the signaling of this receptor (Chermnykh et al., 2018). Several growth factors secreted by MSCs are deposited in the ECM and are activated upon tissue damage. Vesicles associated with the matrix have also been found in the ECM produced by MSCs, but their impact on the regenerative effects of MSCs yet remains to be investigated (Huleihel et al., 2016; Novoseletskaya E. et al., 2019). Therefore, filling the ECM with biologically active molecules or exposition of active molecules, as well as its ability to act as a ligand, can provide fine regulation of stem cell behavior during tissue repair and localize them in the vicinity of MSCs, making it possible to receive paracrine signals of a different nature.

Individual ECM components produced by MSCs can mimic the effects of growth factors by acting as agonists or antagonists of receptor tyrosine kinases. MSCs, mesenchymal stromal cells; ECM, extracellular matrix.

#### Effects of Secreted Factors, Extracellular Vesicles, and Organelle Transfer

**Figure 3:**
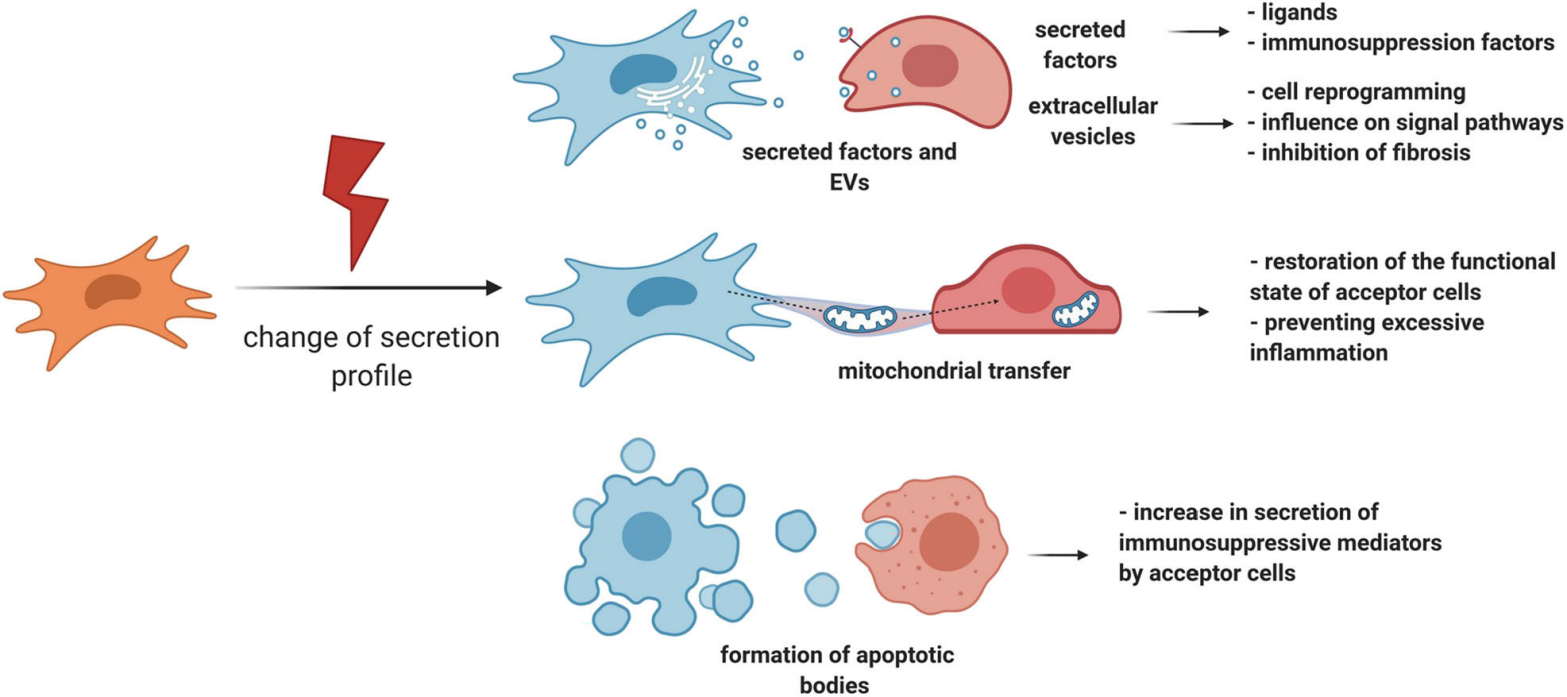
MSC can regulate tissue regeneration by secretion of paracrine factors, extracellular vesicles, and organelle transfer. MSCs have the ability to respond to damage by changing the number and panel of secreted factors. Particularly, MSCs can exhibit a paracrine response to stem cell demand signals during damage and contribute to tissue regeneration. Another important response of MSCs to damage is the activation of an immunosuppression program. In addition to the secretion of individual paracrine factors, MSCs can secrete EV or transfer individual organelles, such as mitochondria, through vesicular or tunnel nanotube transport. MSCs, mesenchymal stromal cells; EV, extracellular vesicles.

MSCs have the ability to respond to damage through other manners, for example, by changing the number and panel of secreted factors (Caplan and Correa, 2011; Kalinina et al., 2015a) (Figure 3). This may also be one of the strategies to regulate the behavior of stem cells in a niche, and ultimately tissue regeneration. Therefore, when a small intestine crypt stem cell is damaged, the population of GLI1-positive MSCs becomes the primary source of ligands for the Wnt signaling pathway, while normal secretion of this ligand is also provided by Paneth cells. Impaired secretion of Wnt ligands is accompanied by an increased expression of intestinal epithelium Shh ligands, which can serve as stimulatory signals for the proliferation of GLI1-positive MSCs. Consequently, MSCs can exhibit a paracrine response to stem cell demand signals during damage and contribute to tissue regeneration (Degirmenci et al., 2018).

Another important response of MSCs to damage is the activation of an immunosuppression program. Therefore, proinflammatory cytokines accumulating in the lesion area, such as TNF-alpha, IL-1-alpha, and IL-1-beta, trigger the expression of immunosuppression mediators such as COX2, PGE2, and IDO by MSCs. These molecules can inhibit excessive tissue damage caused due to the effects of proinflammatory cytokines, thereby positively contributing to regeneration (Galipeau et al., 2016; Gomez-Salazar et al., 2020). In particular, IDO can mediate the therapeutic effects of MSCs, which was reflected by faster tissue repair as well as its protection against structural changes and cell damage in the mouse hind limb ischemia-reperfusion model (Masoumy et al., 2014). It is noteworthy that MSCs can trigger the synthesis of immunosuppression mediators in target cells as well. One of the mechanisms may be the initiation of apoptotic processes in MSCs in the proinflammatory microenvironment (Solodeev et al., 2018), because the absorption of apoptotic MSCs by macrophages leads to the secretion of IDOs (Galleu et al., 2017).

In addition to the secretion of individual paracrine factors, MSCs can secrete EV, mostly exosomes (Basalova et al., 2020), whose composition also varies depending on external signals (Lopatina et al., 2014). The significance of this cell communication mechanism is crucial because MSCs can transmit molecules of various nature, including proteins, lipids, and nucleic acids such as mRNA and regulatory non-coding RNAs, within EV to target cells (Kalinina et al., 2015b; Yáñez-Mó et al., 2015; Efimenko et al., 2016; Basalova et al., 2020). Due to the possible targeted effects of EV, their transfer to nearby cells can help fine-tune the effects of the MSC secretome (Hoshino et al., 2015). The EV secreted by MSCs contain a large number of micrornas that are capable of inhibiting the translation of mRNA in target cells both *in vitro* and *in vivo* (Friedman et al., 2009; Wahid et al., 2010). Among the most represented in the MSC-produced EV, microRNAs were found to regulate the maintenance of the stem cell pool by changing the expression of the components of the Wnt, PDGF, and TGF-beta signal transmission pathways. The EV secreted by MSCs can also contribute to tissue regeneration due to their effect on the microenvironment. In particular, studies have demonstrated that they contain micrornas that suppress the formation of myofibroblasts and, accordingly, the development of fibrosis by suppressing the TGF-beta2/SMAD2 pathway and the production of ECM proteins (Fang et al., 2016; Basalova et al., 2020).

Using EV, MSCs can transmit mRNAs that are translated in the corresponding functional proteins in target cells, perform an immunosuppressive function, and alter the proliferation of target cells (Ragni et al., 2017). The literature reports evidence of the transfer of growth factor molecules in EV that stimulate the proliferation of target cells during *in vitro* damage (Tomasoni et al., 2013), as well as some transcription factors, which indicates a possible protective function of this mechanism.

However, MSC signaling is not limited to paracrine factors or complexes of regulatory molecules in EV. MSCs can mediate tissue regeneration by transferring individual organelles, such as mitochondria, through vesicular or tunnel nanotube transport. Mitochondrial transfer can be induced by signals from damaged cells, DAMPs, in the form of mitochondrial DNA, mitochondrial proteins, or whole mitochondria (Mahrouf-Yorgov et al., 2017). MSCs, in turn, transfer their own mitochondria to their microenvironment cells, which can lead to restoration of the functional state of acceptor cells and protection of the niche stem cell pool from depletion, respectively. Therefore, in a model of acute lung damage, it was demonstrated that the transfer of mitochondria from MSCs to alveolar epithelial cells reduces the negative consequences of acute lung damage through normalization of the functions of alveolar epithelial cells (Islam et al., 2012). In contrast, through the transfer of mitochondria, MSCs can also restrain the effects of microenvironment cells, preventing excessive inflammation. Hence, MSCs inhibit the secretion of proinflammatory cytokines by activated macrophages and stimulate their phagocytic function (Morrison et al., 2017). *In vivo*, these effects can limit the excess damage caused due to the proinflammatory microenvironment, as well as slow the spread of proinflammatory signals due to their phagocytosis by macrophages.

As not only local but also systemic stimuli are involved in the regulation of regeneration, it is reasonable to consider the ability of MSCs to respond to systemic signals, as well as modulate them. Consequently, nestin-positive MSCs associated with adrenergic fibers in response to the stimulation of their own beta-3 adrenergic receptors reduce the expression of hematopoiesis maintenance genes (Méndez-Ferrer et al., 2010). These effects can be a part of a program for preserving the stem cell pool by redistributing them into intact niches, as indicated by the need for a functioning sympathetic nervous system to restore bone marrow in damage models induced by genotoxic agents. An alternative assumption may be their involvement in the effects of the sympathetic nervous system and other receptors such as beta-2 (Lucas et al., 2013). In addition to sympathetic innervation, MSCs can regulate the behavior of hematopoietic progenitor cells in a paracrine manner in response to neuropeptide signals as well (Rameshwar and Gascón, 1996).

Through paracrine exposure, MSCs can modulate central nervous system signals by regulating tissue regeneration. Therefore, due to their immunomodulatory properties, MSCs can change the local level of cytokines that are capable of triggering the transmission of inflammatory reflex by the afferent vagus nerve. The launch of this reflex can lead to the suppression of excess inflammation that adversely affects tissue regeneration (Rosas-Ballina et al., 2008; Pavlov and Tracey, 2017). Altogether, MSCs can transmit paracrine signals from the nervous system, as well as modulate its function, which can mediate tissue regeneration.

### MSCs Regulate Tissue Regeneration by Restoring the Cellular Components of a Niche

In addition to the paracrine potential involved in tissue regeneration, MSCs can act as precursors of individual niche components. In particular, during bone restoration after a fracture, a soft bone callus consisting of fibroblasts and chondrocytes is formed that ensures the mechanical stability of the bone in the fracture area. Bone immobilization occurs due to the secretion of ECM by chondrocytes, which can be derived from MSCs. In the later stages of repair, primary bone formation occurs. The major participants in this process are osteoblasts, whose precursors can also be MSCs (Knight and Hankenson, 2013). Lineage tracing studies have demonstrated that adipose tissue turnover and recovery after damage are also provided by the differentiation of PDGFRb-positive mural cells that are considered as MSCs (Vishvanath et al., 2016). It is also known that MSCs are used in the therapeutic restoration of adipose tissue damaged after surgery or injury (Choi et al., 2010).

It should be noted that the direction of differentiation of MSCs determines the ability of a niche to support stem cells. Therefore, the imbalance between osteogenic and adipogenic differentiation of MSCs leads to a decrease in the pool of osteoblasts, which play a vital role in maintaining hematopoiesis (Justesen et al., 2001; Visnjic et al., 2004). It has been demonstrated that MSCs have the potential for differentiation into other cells. Hence, when simulating chronically injured hearts, MSCs transplanted to the lesion site remained in the donor organism for a long time and expressed the transcription factors of cardiomyocytes, as well as the markers of endothelial cells and vascular smooth muscle (Quevedo et al., 2009).

Apparently, MSCs can replenish the pool of niche cells in methods other than differentiation. This mechanism may be especially important in the case of terminally differentiated cells that do not proliferate in the niche. Sertoli cells that are essential for the normal function of the SSC niche can be noted among these cells. Therefore, injecting the MSC secretome can help attract lost Sertoli cells from the pools of progenitor cells. This assumption was indirectly supported by the restoration of the Sertoli cell pool without visible proliferation at the observed control points *in vivo*, as well as the stimulation of Sertoli cell migration by MSC secretion *in vitro* (Figueiredo et al., 2016; Sagaradze et al., 2019a).

Despite the significant regenerative potential of MSCs, the stem cell pool in the tissue can be completely lost in the damaged tissue. However, some cells of an adult organism have the potential of plasticity and can acquire a stem cell phenotype, fill a niche, and restore a lost organ or tissue (Rompolas et al., 2013). Presumably, in case of damage to individual stem cell niches, MSCs can participate in the transfer of stem cell properties to differentiated cells. In particular, MSCs, being a subpopulation of intestinal mesenchymal cells, can secrete proinflammatory cytokines, for example, IL-11, in response to tissue damage (Thomson et al., 2020). Recently it was demonstrated that when the intestinal crypt was damaged, the stem cell pool was restored primarily due to the differentiation of absorptive and secretory progenitors, and Ascl2 transcription factor was required for restoring the ISC pool. Among the list of genes regulated by Ascl2, a receptor for IL-11 was found, and the introduction of recombinant IL-11 increased the regenerative potential of crypt cells (Murata et al., 2020).

It is noteworthy that MSCs can not only stimulate the acquisition of stem cell properties by niche cells but also give rise to stem and progenitor cells, which ensures the restoration of the pool of supporting niche cells. Thus, restoration of the Leydig cell pool was detected in a mouse model of damage to Leydig cells with ethane methane disulfonate (EDS). In the early stages of observation itself, the restoration of the Leydig cell pool was accompanied by an increase in the number of nestin-producing vascular smooth muscle cells, as well as pericytes in the testis, compared to that in the testes of intact animals.

After modeling damage, the nestin-positive cells left the walls of blood vessels, acquired steroidogenic properties, and lost the nestin expression. The conversion into steroidogenic cells was also accompanied by the loss of alpha-SMA, which indicated the possibility of transdifferentiation of pericytes into progenitor Leydig cells (Davidoff et al., 2004). The possibility of replenishing the Leydig stem cell pool by the transdifferentiation of MSCs was indirectly confirmed by the greater proximity of the Leydig stem cell transcript to MSCs (Stanley et al., 2011). Furthermore, when individual types of MSCs were transplanted into a rat testicle lacking Leydig cells due to EDS treatment, the Leydig cell population was restored. Consequently, 21 days after the transplantation, the researchers observed higher blood testosterone levels in the MSC group than in the control group. Remarkably, some transplanted cells expressed steroidogenesis markers that are generally expressed by Leydig cells (Zhang et al., 2016). However, MSCs isolated from some other tissues during transplantation into the testis may not participate in the formation of Leydig cell populations during restoration, which may further support the tissue specificity of some functions of MSCs (Curley et al., 2019) and suggest analyzing the possibility of transdifferentiation of MSCs from the testicle into Leydig cells.

Accumulating evidence indicates that MSCs, activated by damage stimuli, can provide a stem cell niche with multiple signals aimed at restoring complicated cell-to-cell communications. As mentioned earlier, MSCs possess immunomodulatory properties and regulate the complex balance between different subtypes of immune cells. Widely recognized as proangiogenic, these cells also produce abundant amounts of antiangiogenic factors, such as pigment epithelial-derived factor and thrombospondins, and could be modified by specific stimuli to limit new blood vessel formation and rather stabilize the vascular structures (Lopatina et al., 2014). In tissue-specific stem cell niches, MSCs might provide regulatory factors restricting the injury-induced responses of niche cells. Hence, we suppose that the ability of MSCs to restore the SSC niche due to the effects of their secretome, which we observed in a rat model of abdominal cryptorchidism (Sagaradze et al., 2019a), could be at least partially mediated by secreted insulin-like growth factor-binding proteins (IGFBPs) (Kalinina et al., 2015a). Niche damage causes hyperplasia of the interstitial compartment where Leydig cells localize and produce insulin-like growth factor 1 (IGF1) that is critical for the functioning of both SSCs and supporting cells. Importantly, excessive amounts of IGF1 could predominantly stimulate spermatogonial differentiation leading to the depletion of undifferentiated SSCs and block in spermatogenesis in the subsequent cycles (Safian et al., 2016). IGF1 and other IGFs bind to IGFBPs with greater affinity than they bind to their receptors (Youssef et al., 2017), which allows to protect undifferentiated spermatogonia in the testis against excessive differentiation. Furthermore, recent discoveries have identified that IGFBP-3 plays a dual function of a gatekeeper (induction of apoptosis and cell cycle arrest) and a caretaker (DNA repair through interaction with DNA-PK, induction of autophagy by interaction with GRP78, and the ability to regulate sphingolipids required for the cell survival pathways) through mechanisms independent of IGFs (Varma Shrivastav et al., 2020). Taken together, by secreting IGFBPs, MSCs might be able to correct the imbalance of IGF1-mediated regulatory effects between cells within the niche, including Leydig cells, Sertoli cells, and SSCs.

## Senescence of MSCs Contributes to Alterations of Tissue Regeneration

Senescence, a cellular response to endogenous and exogenous stresses limiting the proliferation of damaged and dysfunctional cells, markedly affects the processes of tissue homeostasis and regeneration and contributes to both physiological aging and age-related diseases (van Deursen, 2014; Childs et al., 2015; McHugh and Gil, 2018). Cell senescence can be induced by harmful stimuli such as DNA damage, telomere shortening, oncogenic insults, metabolic stress, epigenetic changes, and mitochondrial dysfunction (Liu et al., 2020). Senescent cells accumulate with aging in different tissues, and stem cell aging and replicative exhaustion are considered as hallmarks and promoters of aging and functional attrition in organisms (van Deursen, 2014; Childs et al., 2015).

There is a vast amount of evidence indicating the important associations between tissue injury, especially chronic injury, and accumulation of senescent cells, including those in MSC populations (Childs et al., 2015; Cárdenes et al., 2018; Neri and Borzì, 2020). The key regulators of MSC senescence remain still incompletely identified and can represent therapeutic targets to counteract age-associated diseases and organismal aging. MSC senescence is accompanied by functional alterations that caused due to metabolic, genetic, epigenetic, transcriptional, and translational changes (see the detailed review by Neri and Borzì, 2020). In addition, these cells acquire a senescence-associated secretory phenotype (SASP) involving the secretion of factors that can affect the behavior of neighboring cells via autocrine/paracrine mechanisms and reprogram the microenvironment toward the prosenescent state (Borodkina et al., 2018; Campisi et al., 2019). During aging of an organism, senescent MSCs imply an impairment of stem cell functions contributing to the progressive decrease in tissue maintenance and regeneration, because the regenerative potential of MSCs essentially declines with age together with an increase in senescence markers (Stolzing et al., 2008; Schimke et al., 2015; Yang et al., 2018; Khong et al., 2019; Neri and Borzì, 2020). Hence, the delay in fracture healing with advanced age is attributed to a decreased number and function of MSCs (Wagner et al., 2019). *In vivo*, MSC senescence implies reduced osteogenic capacity, thereby contributing to age-related diseases such as osteoporosis. It has been demonstrated that miR-1292 could positively regulate MSC senescence through the wingless-related integration site (Wnt) /β-catenin signaling pathway and by targeting frizzled 4 receptor (FZD4), thereby emerging as a potential target to treat osteoporosis (Fan et al., 2018). Furthermore, Liu et al. (2015) showed that the loss of osteogenic potential in aged bone marrow-derived MSCs is mediated by p53 increase through the miR-17 pathway (Liu et al., 2015).

MSCs derived from aged donors exhibit impaired ability to stimulate vascularization due to the reduced secretion of proangiogenic factors—including vascular endothelial growth factor, placental growth factor, and hepatic growth factor (HGF)—whereas there is an increased secretion of antiangiogenic factors such as thrombospondin-1 (TBS1), plasminogen activator inhibitor-1 (PAI-1), and certain factors involved in ECM remodeling (Efimenko et al., 2011, 2014; Khan et al., 2011).

Aged MSCs have a diminished capacity to inhibit the proliferation of allogeneic peripheral blood mononuclear cells compared to that of younger cells (Gnani et al., 2019). The production of SASP factors such as interleukin 6 (IL-6), IL-8, and monocyte chemotactic protein 1 (MCP-1) in the conditioned medium of senescent MSCs was found to be increased compared to that in young MSCs, which not only drives responses that reinforce senescence in a cell-autonomous manner but also acts on neighboring cells via a paracrine mechanism to accelerate senescence (Gnani et al., 2019).

An earlier study demonstrated the critical role of EV and non-coding RNAs in their composition in the regulation of target cells by MSCs during aging (Xu and Tahara, 2013). Consequently, Kulkarni et al. (2018) showed that certain miRNAs within exosomes secreted by young MSCs can suppress cell aging of hematopoietic stem cells, whereas vesicles from senescent MSCs significantly aggravated this process (Kulkarni et al., 2018).

It is important to note that MSCs could also acquire the senescent phenotype and properties in different diseases without the direct relationship with advanced age (Dzhoyashvili et al., 2014; Cárdenes et al., 2018). As an example, MSCs derived from the adipose tissue of obese subjects were found to have lower self-renewal properties because of increased oxidative and metabolic stress affecting mitochondria, thereby leading to DNA damage, telomere shortening, reduced proliferation and stemness, increased apoptosis, and senescence (Pérez et al., 2015). Perhaps, the accumulation of senescent MSCs is likely a response to the damage stimuli such as chronic inflammation, and these changes are not certainly negative. Consequently, several studies have demonstrated the benefits of senescent cells in wound healing, injury repair, and tissue regeneration (Ritschka et al., 2017; Campisi et al., 2019). However, the pathological effects begin to prevail along with their accumulation and prolonged persistence in tissues. As a result, the accumulation of senescent MSCs could lead to the disruption of their regulatory function, mediated primarily through the effects of their secretome, and to the pathological remodeling of their microenvironment, which would ultimately attenuate the regenerative potential of tissues.

## Concluding Remarks: Reconsidering the Therapeutic Use of MSCs

Most of the therapeutic effects of MSCs are aimed at maintaining stem cells after injury, as well as creating the infrastructure for restoring the stem cell niche. MSCs have the ability to regulate niche restoration, focusing on signals of tissue damage, as well as nearby cells and the nervous system. Presumably, the ability of these cells to secrete regulatory molecules and complexes of various nature plays a key role in regulating the function of MSCs. However, the contribution of MSC differentiation to niche restoration can also be significant, because an imbalance in differentiation leads to a disruption in the cellular composition of the stem cell niche and regulation of stem cell behavior. The potential of MSCs to develop into tissue-specific stem cells, as well as to support the dedifferentiation of microenvironment cells into stem cells, is also important.

MSCs are actively involved in tissue regeneration, which is reflected by the effectiveness of using MSCs or MSC-derived products in several injury models. This is due to the presence of common mechanisms of tissue regeneration and common functional patterns of MSCs that can affect them (Strioga et al., 2012). However, the source of MSCs can to some extent determine the effectiveness of using MSCs or MSC-derived products. One of the reasons may be the different sensitivity of MSCs to culture conditions. Hence, cell culture media can exert different effects on the secretory or differentiation potential of individual types of MSCs (Al-Saqi et al., 2014; Sagaradze et al., 2019a). Another reason may be the persistent specialization of MSCs with respect to tissue-specific stem or resident cells (Tran et al., 2008; Pittenger et al., 2019). Therefore, it is advisable to consider the use of MSCs from tissue-matched sources to increase the effectiveness of MSCs or MSC-derived products (Niemeyer et al., 2010).

Senescence of MSCs causes functional changes and impairment of their regenerative capacity emphasizing the importance of potential rejuvenation strategies, especially for autologous MSC-based therapy (Efimenko et al., 2015; Neri and Borzì, 2020). However, the current knowledge of senescence is primarily based on bulk cell data. Novel techniques such as single-cell RNA sequencing, extended time-lapse *in vivo* imaging, and genetic lineage tracing would provide a more complete understanding of MSC aging process, making it possible to slow senescence or even rejuvenate aged MSCs (Liu et al., 2020).

If it is impossible or technologically complex to implement this approach, one can consider alternative methods for restoring a stem cell niche using MSCs. Therefore, it is possible to activate MSC-mediated tissue regeneration using mediators of inflammation and regeneration *in vivo* (Van Megen et al., 2019). Activation of MSCs can also be facilitated by simulating positive feedback between MSCs and resident cells (Ferland-McCollough et al., 2018). Hence, the most effective use of MSCs in regenerative medicine can be achieved if MSCs are able to transduce the signals, and target niches will perceive tissue-specific proregenerative signals. Considering MSCs as critical contributors that preserve a pool of stem cells and restore a stem cell niche after injuries, we conclude that for an effective stimulation of tissue regeneration, it is extremely important to understand how to manage the tissue-specific interactions between MSCs and niche cells.

## Author Contributions

GS, AE, and VT: conceptualization. GS, NB, AE, and VT: writing and editing. All authors have read and agreed to the published version of the manuscript.

## Conflict of Interest

The authors declare that the research was conducted in the absence of any commercial or financial relationships that could be construed as a potential conflict of interest.
